# In ®Entresto we trust

**DOI:** 10.1186/s40959-020-00083-7

**Published:** 2020-11-05

**Authors:** Massimiliano Camilli, Marco Giuseppe Del Buono, Pierantonio Menna, Giorgio Minotti

**Affiliations:** 1 Department of Cardiovascular and Thoracic Sciences, Catholic University of the Sacred Heart, Rome, Italy; 2 Department of Medicine, Center for Integrated Research and Unit of Drug Sciences, Campus Bio-Medico University, Via Alvaro Del Portillo 21, 00168 Rome, Italy

**Keywords:** Sacubitril/valsartan, Heart failure, Cardio-oncology

## Abstract

Sacubitril/Valsartan (S/V) is a novel and remarkably effective opportunity to treat heart failure with reduced ejection fraction (HFrEF). However, patients with HFrEF induced by cancer therapy were a priori excluded from the registration study. The value of S/V in this important subgroup of patients needs to be firmly established. In this issue of *Cardio-Oncology*, Gregorietti et al. report on the effects of S/V in a small group of cancer patients, primarily women with breast cancer treated with anthracyclines. The data are limited but seem to confirm the encouraging results of prior studies, paving the way to foster the use of S/V in cardio-oncology patients and hopefully, to design ad hoc prospective studies in this highly vulnerable population.

In this issue of *Cardio-Oncology*, Gregorietti et al. [1] report on the clinical effects of Sacubitril/Valsartan (S/V,®Entresto) in a small group of cancer patients, primarily women with breast cancer who developed Heart Failure with reduced Ejection Fraction (HFrEF) after treatment with anthracycline and, in few cases, with anti-ErbB2 antibodies, like trastuzumab and pertuzumab.

S/V has recently emerged as a game-changer in the treatment of HFrEF. The PARADIGM-HF trial [2] (Prospective Comparison of ARNI with ACEi to Determine Impact on Global Mortality and Morbidity in Heart Failure) showed that S/V was superior to enalapril in reducing cardiovascular mortality (13.3% vs 16.5%; HR, 0.80 [95%CI, 0.71–0.89]) and rate of hospitalization for HF (12.8% vs 15.6%; HR, 0.79 [95% CI, 0.71–0.89]) in patients with chronic HFrEF. These findings were then extended to the acute setting in the PIONEER-HF trial [3] (Comparison of Sacubitril-Valsartan vs Enalapril on Effect on NT-proBNP in Patients Stabilized from an Acute Heart Failure Episode), which included hemodynamically stable patients who were admitted to the hospital with a primary diagnosis of acute decompensated HF. Over a follow-up period of 8 weeks, S/V, compared to enalapril, resulted in a greater reduction in NT-proBNP (− 46.7% vs − 25.3%; ratio of change 0.71 [95% CI, 0.63–0.81]) and greater reduction in hospitalization for heart failure (8.0% vs 13.8%; HR, 0.56 [95% CI, 0.37–0.84]). The TRANSITION study [4] (Comparison of Pre- and Post-discharge Initiation of LCZ696 Therapy in HFrEF Patients After an Acute Decompensation Event) evaluated the safety and efficacy of in-hospital versus post-discharge initiation of S/V in patients with acute heart failure (AHF) and found in-hospital initiation to be feasible and well-tolerated.

The benefits of S/V in chronic HFrEF may also extend to patients beyond those studied in the PARADIGM-HF trial. The PROVE-HF study [5] (Prospective Study of Biomarkers, Symptom Improvement and Ventricular Remodeling During Entresto Therapy for Heart Failure) found that the magnitude of improvement in indices of cardiac structure and function was consistent across subgroups that were not represented in the PARADIGM-HF trial [2] (namely those with NT-proBNP levels lower than those determined by the entry criteria for PARADIGM-HF [2], those not achieving the target S/V dose, and those with new-onset HF or naïve to ACE inhibitor and ARB). Main outcomes and features of the aforementioned studies are summarized in Table 1.

**Table 1:** Main characteristics, end points and outcomes of Sacubitril/Valsartan trials of non-oncologic patients with heart failure^a^

Clinical trial	No. of patients	Follow-up (months)	End point	Event rate (%)		HR (95% CI)	*P* value
				Intervention	Control		
**PARADIGM-HF**	8442	27	All-cause mortality	17.0	19.8	0.84 (0.76–0.93)	<.001
			CV death, HF hospitalization	21.8	26.5	0.80 (0.73–0.87)	<.001
			HF hospitalization	12.8	15.6	0.81 (0.71–0.89)	<.001
**PIONEER-HF**	881	2	HF hospitalization	8.0	13.8	0.56 (0.37–0.84)	–
			All-cause mortality	2.3	3.4	0.66 (0.30 to 1.48)	–
			NT-proBNP reduction	46.7	25.3	0.71^b^ (0.63–0.81)	< 0.001
**TRANSITION**	1002	2.5	Proportion of patients attaining 97/103 mg bid target dose after 10 weeks	45.4	50.7	0.90^c^ (0.79–1.02)	0.099
**PROVE-HF**	654	12	Correlation between log2–NT-proBNP changes in patients treated with S/V and changes in measures of cardiac volume and function	LVEF (*r* = −0.381 [IQR, −0.448 to −0.310]; *P* < .001), LVEDVI (*r* = 0.320 [IQR, 0.246 to 0.391]; *P* < .001), LVESVI (*r* = 0.405 [IQR, 0.335 to 0.470]; *P* < .001), LAVI (*r* = 0.263 [IQR, 0.186 to 0.338]; *P* < .001), E/e′ ratio (*r* = 0.269 [IQR, 0.182 to 0.353]; *P* < .001).			

*CI* confidence interval, *CV* cardiovascular, *LAVI* left atrial volume index, *LVEDVI* Left ventricle end-diastolic volume index, *LVEF* left ventricular ejection fraction, *LVESVI* Left ventricle end-systolic volume index, *NT-proBNP* N-terminal prohormone of brain natriuretic peptide, *HF* heart failure, *HR* hazard ratio, *IQR* interquartile range, *MO* months, *PARADIGM-HF* Prospective Comparison of ARNI with ACEi to Determine Impact on Global Mortality and Morbidity in Heart Failure, *PIONEER-HF* Comparison of Sacubitril-Valsartan vs Enalapril on Effect on NT-proBNP in Patients Stabilized from an Acute Heart Failure Episode, *PROVE-HF* Prospective Study of Biomarkers, Symptom Improvement and Ventricular Remodeling During Entresto Therapy for Heart Failure, *S/V* Sacubitril/Valsartan, *TRANSITION* Comparison of Pre- and Post-discharge Initiation of LCZ696 Therapy in HFrEF Patients After an Acute Decompensation Event

^a^Based on references [2–5]

^b^Ratio of change

^c^Risk ratio

Thus, a broad spectrum of patients seems to benefit from S/V in terms of reduced morbidity and mortality. Regrettably, however, a history of chemotherapy-related HF over the last 12 months was defined as an exclusion criterion from the PARADIGM-HF trial [2]. This may have been caused by concerns about cancer progressing and requiring second- or third-line oncologic therapies during the course of the trial, which may confound an interpretation of cardiac events adjudication and trial outcomes. Furthermore, whereas an effect of HF drugs on cancer mortality has not been demonstrated [6], risk of oncologic under-treatment might occur if studies do not include an adaptive design that incorporates the unique characteristics and demands of cancer patients. There is an obvious need for clinical trials to include an adequate number of cancer patients with HF, or better still, new HF drugs should be probed in parallel trials in non-oncologic and cancer populations separately.

Nearly each class of cancer drugs has long been known to cause cardiotoxicity, with variable rates and clinical phenotypes of cardiac events [7, 8]. Cancer therapy-related cardiac dysfunction (CTRCD) may in some case be associated with poor prognosis, cardiovascular morbidity and mortality [7, 8]. In particular, anthracycline-related cardiomyopathy manifests as a canonical systolic dysfunction that in its most serious form progresses to HFrEF [7, 8]. Probing S/V in these settings has become an intuitive therapeutic opportunity. S/V does in fact combine the beneficial effects of valsartan on inhibiting neuro-hormonal activation [8] with the action of neprilysin on preventing natriuretic peptides degradation, thus amplifying the effects that such peptides may have on improving the dynamics of myocardial contraction-relaxation [9]. However, the efficacy and safety of S/V in the settings of CTRCD is anecdotal at this point in time, with the available information mostly deriving from case reports [10, 11] or retrospective analysis of relatively few patients.

On the basis of data from a retrospective multicenter registry, Martín-García et al. [12] showed that S/V was well tolerated and could improve myocardial function and structure in 67 cancer patients with CTRCD, mainly in the settings of breast cancer (45%) and lymphomas (39%). Seventy percent of these patients had been treated with anthracyclines and a total of 12% had received the anti-HER2 antibody, trastuzumab. Most of the patients treated with S/V were on anti-HF optimal medical therapy (OMT); baseline NT-proBNP levels, functional class, and left ventricular ejection fraction (LVEF), evaluated by standard echocardiography, markedly improved at a follow-up of 4.6 months. On average, LVEF increased from 33 to 42%. The same authors reported on a group of 10 patients (80% treated with anthracyclines and 10% with trastuzumab), in whom cardiac magnetic resonance (CMR) was performed at baseline and at 3 months follow-up [13]. Results were consistent with S/V reducing LV volumes, improving LVEF (from 35 to 47%) and NTproBNP levels.

In this issue of *Cardio-Oncology* Gregorietti et al. [1] provide further evidence on the efficacy and safety of S/V in the settings of CTRCD and HFrEF. The authors describe the effects of S/V in a prospective cohort of 28 patients matching the clinical cardiovascular characteristics of patients recruited in the PARADIGM-HF trial. Most of the patients were women with breast cancer, mainly treated with anthracycline (82.1%), and were receiving OMT. Median NT-proBNP declined from 997.5 pg/ml (IQR 663.8–2380.8) to 416.5 pg/ml (IQR 192.0–798.2) (*p* < 0.001) at median 20 months follow-up. LVEF increased from 26.7 ± 5.4% to 32.3 ± 5.5% (*p* < 0.001) and there were significant improvements in diastolic left ventricle diameter (from a baseline median of 67.5 mm to 60.0 mm; *p* < 0.001), and in mitral valvular regurgitation, which denoted a favorable impact of S/V on left ventricle remodeling. All patients included exhibited an enhancement in exercise tolerance at follow-up, as indicated by the change in NYHA functional class (at the end of follow-up: 57% of patients were NYHA I and 43% NYHA II) and by median 6-min walking test improvement (from 300 m to 410 m; *p* < 0.001). In terms of safety end-points, there were no differences between basal and follow-up levels of serum creatinine or potassium.

An additional point of consideration pertains to the presence of cardiovascular risk factors (CVRF) in the patients treated with S/V. CVRF cause a remarkable effect on predisposing to, or accelerating the development of CTRCD and HFrEF. The unanswered question is whether cancer patients with CVRF should merit earlier and/or more aggressive pharmacologic interventions than is recommended by international guidelines for the general population [14].

The study by Gregorietti et al. [1] shows some obvious limitations, including the limited sample size, an insufficient characterization of patients’ oncological history, the observational framework of the study design. In spite of these limitations, the study offers one more piece of evidence to improve the clinical management of patients with CTRCD. The patient population was at glance similar to that described in previous cardio-oncology studies of S/V. This might be perceived as a lack of novelty but in fact it helps to strengthen the information on which new HF therapies should rest to gain new clinical indications. In the case of S/V, it is precisely this kind of reports that helps to foresee the benefits a new drug may offer to patients excluded from the registration trials and corollary studies.

CTRCD is an umbrella definition, embracing clinical phenotypes and stages of cardiomyopathy induced by a multitude of agents with different mechanisms of action. Once considered as an intractable disease, anthracycline-related cardiomyopathy is now known to respond to modern HF therapy, particularly when inhibitors of renin-angiotensin system are used following early detection of myocardial injury [15]. It goes without saying that RCTs would be needed not only to confirm the available evidence of S/V efficacy and safety in HFrEF from anthracycline-based therapy, but also to decipher the role of S/V in the settings of early cardiotoxicity or in homogeneous populations treated by agents other than anthracyclines. We hope to see these studies soon,®Entresto is a trustable drug that warrants such opportunities.

## Data Availability

Not applicable.

## Abbreviations

CTRCD: Cancer therapy-related cardiac dysfunction
EF: Ejection fraction
HF: Heart failure
HFrEF: HF with reduced ejection fraction
NYHA: New York Heart Association
NT-proBNP: N-terminal prohormone of brain natriuretic peptide
OMT: Optimal medical therapy
S/V: Sacubitril/Valsartan
